# Feasibility and Functional Outcome of Robotic Assisted Kidney Transplantation Using Grafts With Multiple Vessels: Comparison to Propensity Matched Contemporary Open Kidney Transplants Cohort

**DOI:** 10.3389/fsurg.2020.00051

**Published:** 2020-08-25

**Authors:** Sachin Arakere Nataraj, Feroz Amir Zafar, Prasun Ghosh, Rajesh Ahlawat

**Affiliations:** Institute of Urology and Robotic Surgery Medanta, The Medicity Gurgaon, Gurgaon, India

**Keywords:** robotic assisted kidney transplantation, grafts with multiple vessels, open kidney transplantation, robotic surgery, Vattikuti-Medanta technique

## Abstract

**Introduction:** The aim of the study was to report the perioperative and functional results of Robotic assisted kidney transplantation (RAKT) in Grafts with multiple vessels (GMVs) and compare it to the results of Open kidney transplantation (OKT) with GMVs.

**Materials and Methods:** Patients undergoing RAKT from living donors using GMVs were reviewed from prospectively collected RAKT database at our institution between March 2013 and March 2018. Patient undergoing Open kidney transplantation (OKT) using GMVs served as controls. *Ex-vivo* bench surgical reconstruction of GMVs was done according to specific anatomy. Propensity score matching was used to balance the sample size in the two groups.

**Results:** Of 153 RAKT and OKT procedures, 86 cases were eligible for propensity score matching for the statistically significant variables (standardized difference >0.10) and 43 procedures were assigned to each group. Median anastomoses, total and cold ischemia and rewarming times did not differ significantly between the RAKT and OKT groups. In comparison with OKT in GMVs we found that RAKT with GMVs had less pain score on post op 2nd day (*p* = 0.03). There was also a significant difference in mean analgesic requirement (*p* = 0.02), hospital stay (*p* = 0.05) and incision length (*p* = 0.04). Most of the major, minor surgical, and medical postoperative complications were comparable between the two groups except for wound related events (*p* = 0.002).

**Conclusion:** Multiplicity of renal vessels in RAKT does not adversely affect patient or graft survival compared with the OKT. Satisfactory functional outcome can be achieved by RAKT similar to OKT in GMVs. RAKT seems to have advantage over OKT in that it is less invasive and has the potential to cause fewer low grade complications. Small sample size and short follow-up are the main limitations of the study.

## Introduction

Renal transplantation is the gold standard treatment for patients with end stage renal disease. The main concern is its utilization and outcome in graft with multiple vessels. The vascular surgical techniques involving kidneys with multiple or complicated renal arteries have so advanced recently that most of such grafts can now be engrafted (1–8). Graft with multiple vessels may have poor outcome, especially ureteric complications (9, 10), compared to transplant following single vessels. Multiple vessels have been used in open kidney transplantation (OKT) with comparable outcome despite technical challenges. Studies on Robot-assisted kidney transplantation (RAKT) have shown results similar to OKT with the added benefit of minimal invasive surgery (11, 12). A recent multi-center study in Europe has addressed the feasibility of RAKT in grafts with multiple vessels (GMVs) (13).

Due to lack of evidence on outcomes of RAKT with GMVs, along with limited availability of living donor pool and a very high rate of anatomic variation in renal vasculature, it is important to prioritize the clinical and research studies in this field (11–20). The main objective of this study was to assess whether GMVs may be used in RAKT with outcomes similar to grafts in OKT, using similar surgical reconstruction techniques.

## Materials and Methods

A retrospective review of prospectively maintained database was performed on selected consecutive patients who underwent RAKT with regional hypothermia using GMVs from living donors between March 2013 and March 2018. The study comparing open with RAKT using prospectively maintained database was approved by Medanta IRB on March 13, 2013, with reference number MICR-259/2012. Patients undergoing OKT using GMVs during the same period were used as controls. Grafts that have two or more renal arteries and or renal veins are defined as GMVs.

### Surgical Technique

The surgical team was experienced in open kidney transplantation, living donor nephrectomy, and robotic urologic surgery including robotic transplantation. The grafts were laparoscopically procured from living donors. Grafts was defatted and perfused with cold Ringer's solution for both OKT and RAKT after retrieval.

**Figure 1:**
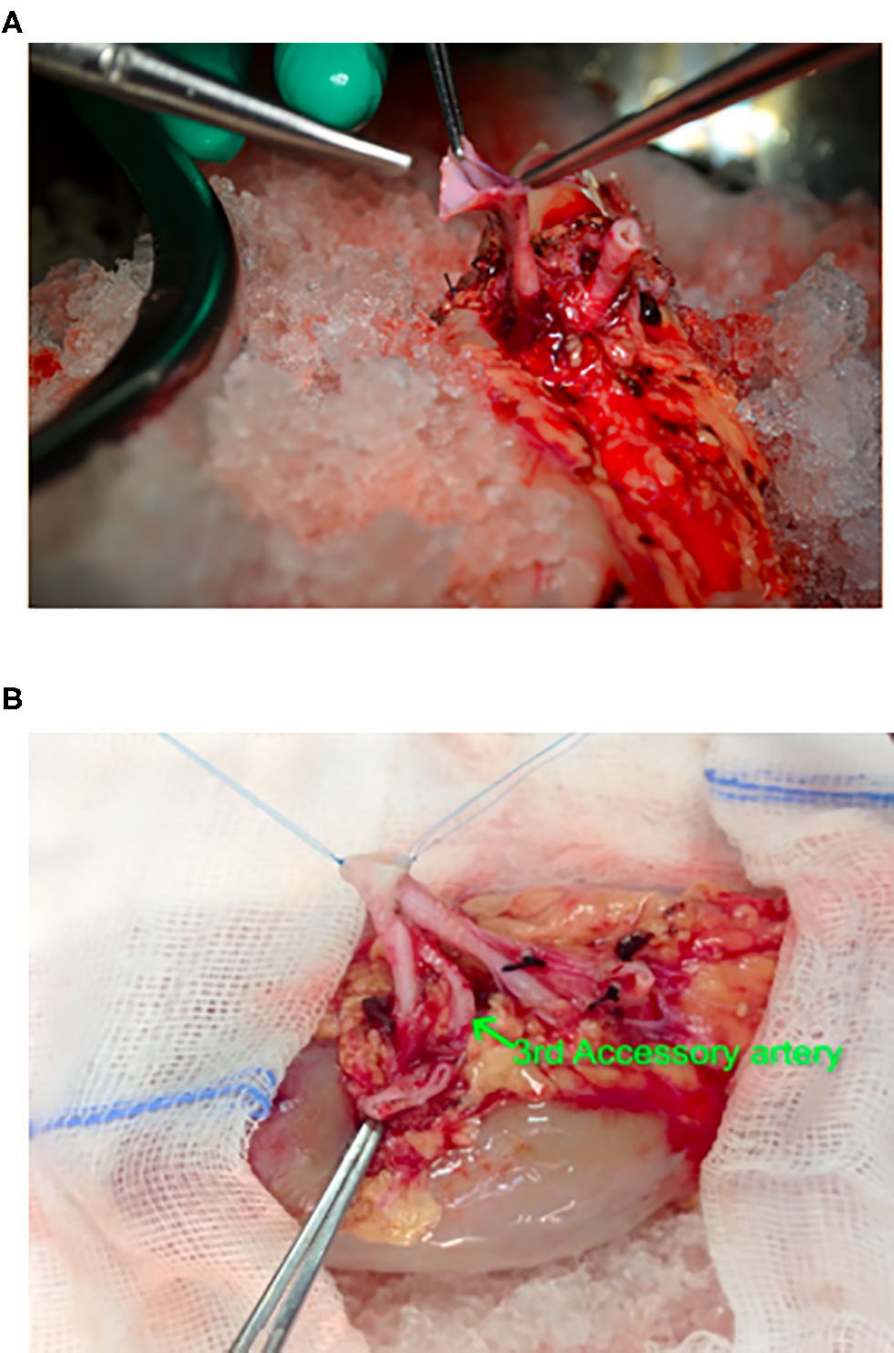
Intraciperative images showing *ex-vivo* bench vascular reconstruction of 3 separate renal arteries, 2 of approximately same calibre from a live related donor. **(A)** Preparation of pantaloon from arteries of same calibre. **(B)** After completion of Pantaloon.

The vascular reconstruction techniques (Table 1 and Figures 1A,B) mirrored those used in historical OKTs at our center. According to the case-specific vascular anatomy, various reconstruction techniques were employed as described previously (13): (1) pantaloon (side-to-side) arterial anastomosis (in a pantaloon fashion); (2) polar artery anastomosis to the inferior epigastric artery; (3) separate arterial anastomoses (end-to-side) to external iliac artery; (4) arterial anastomoses to branches of hypogastric artery; (5) none (if vessel was supplying the upper renal pole with <10% area, ligation of this small accessory artery was performed); (6) pantaloon + anastomoses to inferior epigastric artery if renal arteries >2; and (7) separate venous anastomoses (end-to-side) to external iliac vein.

**Table 1:** Surgical technique for extracorporeal (*ex vivo*) or *in situ* reconstruction for open and robot-assisted kidney transplantation.

**Technique**	**OKT (43)**	**RAKT (43)**
**Grafts with multiple arteries**
Conjoined (side-to-side) arterial anastomosis (*pantaloon* fashion)	17	25
Polar artery anastomosis to the inferior epigastric artery	8	7
Separate arterial anastomoses (end-to-side) to external iliac artery	1	1
Separate arterial anastomoses (end-to-end) to internal iliac artery and (end-to-side) external iliac artery	3	0
Arterial anastomoses to branches of hypogastric artery	0	0
Ligation of small accessory artery, especially if supplying the upper renal pole	3	3
Pantaloon + Anastomoses to inferior epigastric artery	6	5
**Grafts with multiple veins**		
Separate venous anastomoses (end-to-side) to external iliac vein	4	2
None (Ligation of small renal vein)	1	1

The graft kidney with reconstructed vessels was wrapped in a gauze jacket filled with ice slush and introduced into the recipient through open incision or the Gel-point placed via periumbilical incision in RAKT cases. OKT was performed using a conventional modified Gibson incision. In OKT main or pantaloon, artery was anastomosed to internal iliac artery, end to end or external iliac in end to side, and vein to external iliac in end-to-side fashion using 6-0 continuous prolene for artery and 5-0 prolene for vein.

**Figure 2:**
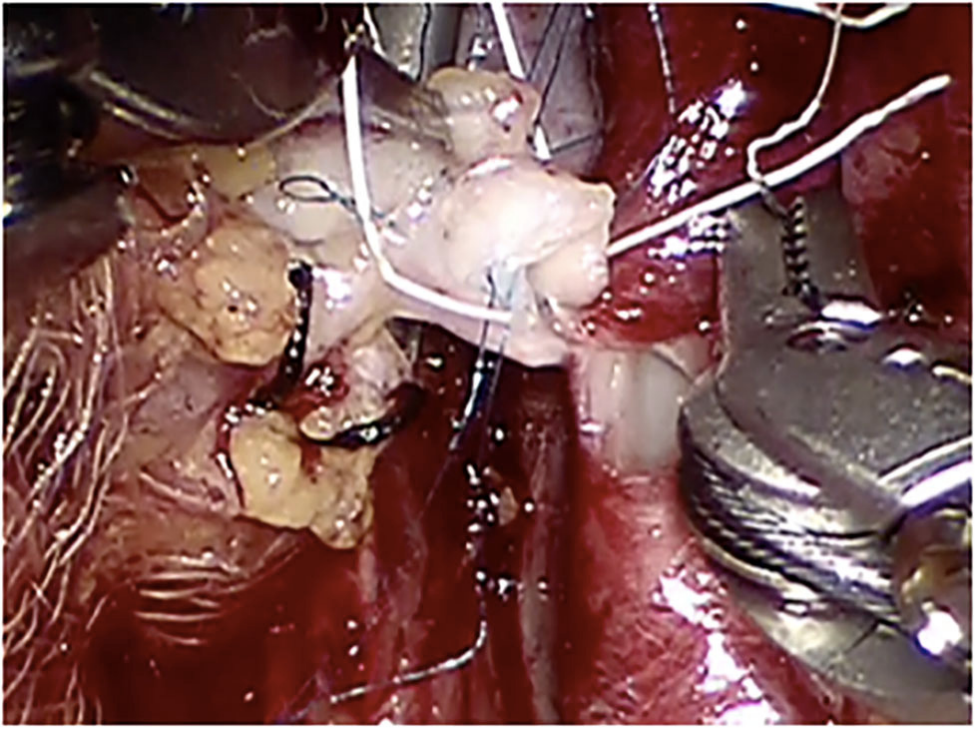
Arterial anastomosis between the reconstructed graft renal artery (side-to-side arterial anastomosis in a pantaloon fashion between the two renal arteries) and the external iliac artery.

**Figure 3:**
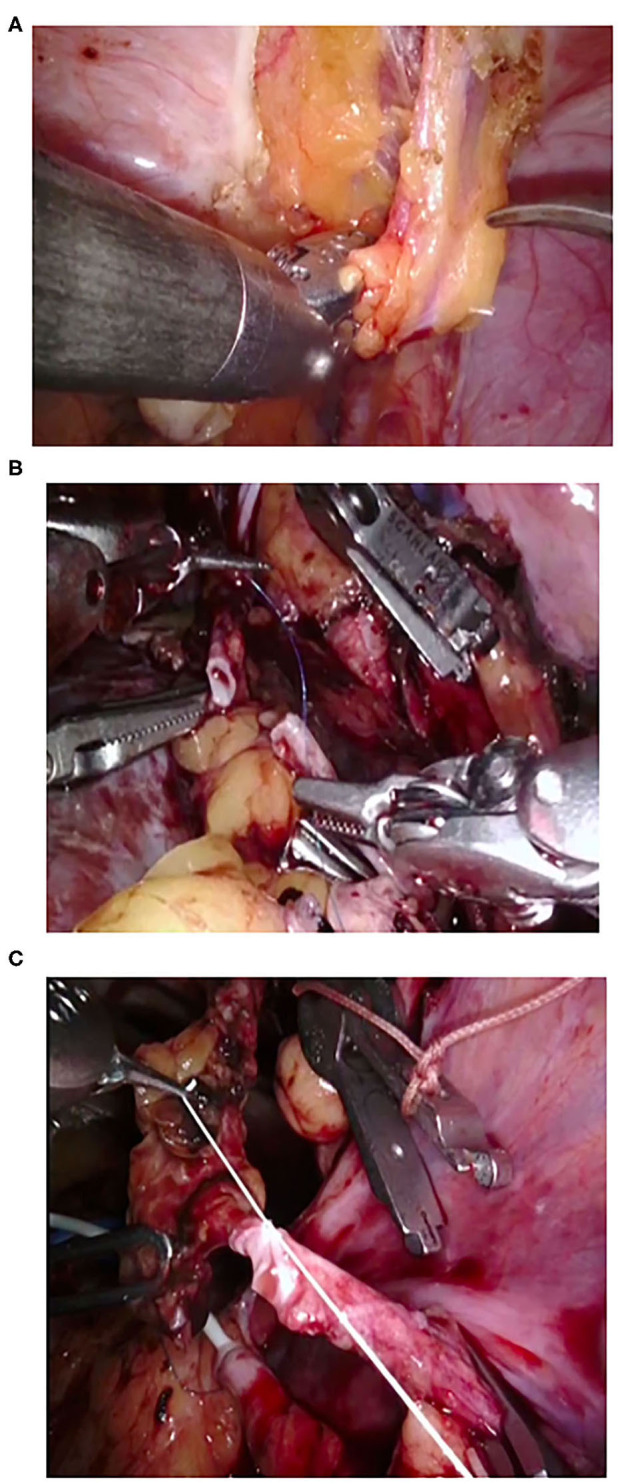
Intraoperative snapshots during robot-assisted kidney transplantation in case of grafts with multiple vessels. **(A)** Preparation of inferior epigastric artery. **(B,C)** Anasturrrosis between inferior epigastric artery and accessory lower pole renal artery.

We performed RAKT using a standardized operative protocol, Vattikuti–Medanta technique (11, 12, 14), as described by us previously. RAKT was performed using the Da Vinci^®^ Xi & Si Surgical System (Intuitive Surgical, Sunnyvale, CA, USA). The patient was placed in 15–30° supine Trendelenburg position. A 5- to 6-cm peri-umbilical incision was made for the placement of a GelPOINT^®^ (Applied Medical, Rancho Santa Margarita, CA, USA). The camera and one assistant port were placed through the GelPOINT^®^ device. Through the right lower quadrant, a 12-mm assistant port and an 8-mm robotic port were inserted. Two more 8-mm ports were inserted into the left lower quadrant. The right external iliac artery and vein were skeletonized, and peritoneal flaps were developed to make room for the harvested kidney. This was followed by preparation of bladder for ureteric anastomosis. Kidney covered with gauze filled with ice slush was placed into the abdomen through the GelPoint. Vessel anastomoses were made after applying two Bulldog clamps. Venous anastomosis was performed followed by arterial anastomosis with 6-0 Gore-Tex sutures (Gore Medical Inc., Flagstaff, AZ, USA) (Figure 2). Arteriotomy was performed by a custom-made arterial punch. The kidney was retroperitonealized and inferior epigastric artery was prepared if additional accessory artery was present in the graft kidney. Anastomosis was performed between accessory artery and inferior epigastric artery with 6-0 Gore-Tex sutures (Figure 3). The ureter was anastomosed using a modified Lich-Gregoir method over a double J stent. Drains were placed in peritoneal cavity at the end of the procedure.

### Definition of Ischemia Times (21):

Warm ischemia time (WIT): Defined as the duration between clamping the graft renal artery and placing the graft kidney on the ice slush.
Cold ischemia time (CIT): Defined as the duration between placing the graft kidney on ice and placing it into the peritoneal cavity.
Re-warming time (RWT): Defined as the duration between placing graft kidney into the peritoneal cavity and perfusing the kidney after vascular anastomosis.
Total ischemia time: Total duration of Warm ischemia time, Cold ischemia time, and Re-warming time.

### Peri-operative Management

Anti-thymocyte globulin/Basiliximab were used as Induction immunosuppressive agents in high-risk patients. Prednisone, tacrolimus, and mycophenolate mofetil were used as maintenance treatment in all transplanted patients. After induction of anesthesia, patients were administered 1 g of intravenous paracetamol. All patients had local anesthetic (0.25% Bupivacaine) injected around the wound during closure. RAKT patients were given PCA morphine only on demand. The OKT patient had in addition an epidural catheter placed for pain relief. Linear visual analog scale was used to assess pain score. Pain scores were recorded at time intervals, viz., 12, 24, and 48 h.

All patients underwent transplant kidney Doppler on day 1. Serum creatinine was performed twice daily for first 3 days, then daily till discharge. Tacrolimus levels in blood were estimated on post-op days 1, 3, and 5 and dose was adjusted accordingly. Drain was removed after post-operative day 2 once drain fluid creatinine was similar to serum creatinine in RAKT cases, but only after drain output was <50 ml in OKTs. Foley catheter was removed on day 5 and DJ stent was removed after 2 weeks. All patients were followed twice a week for the first post-operative month, monthly thereafter for the first year and then 3 monthly.

**Figure 4:**
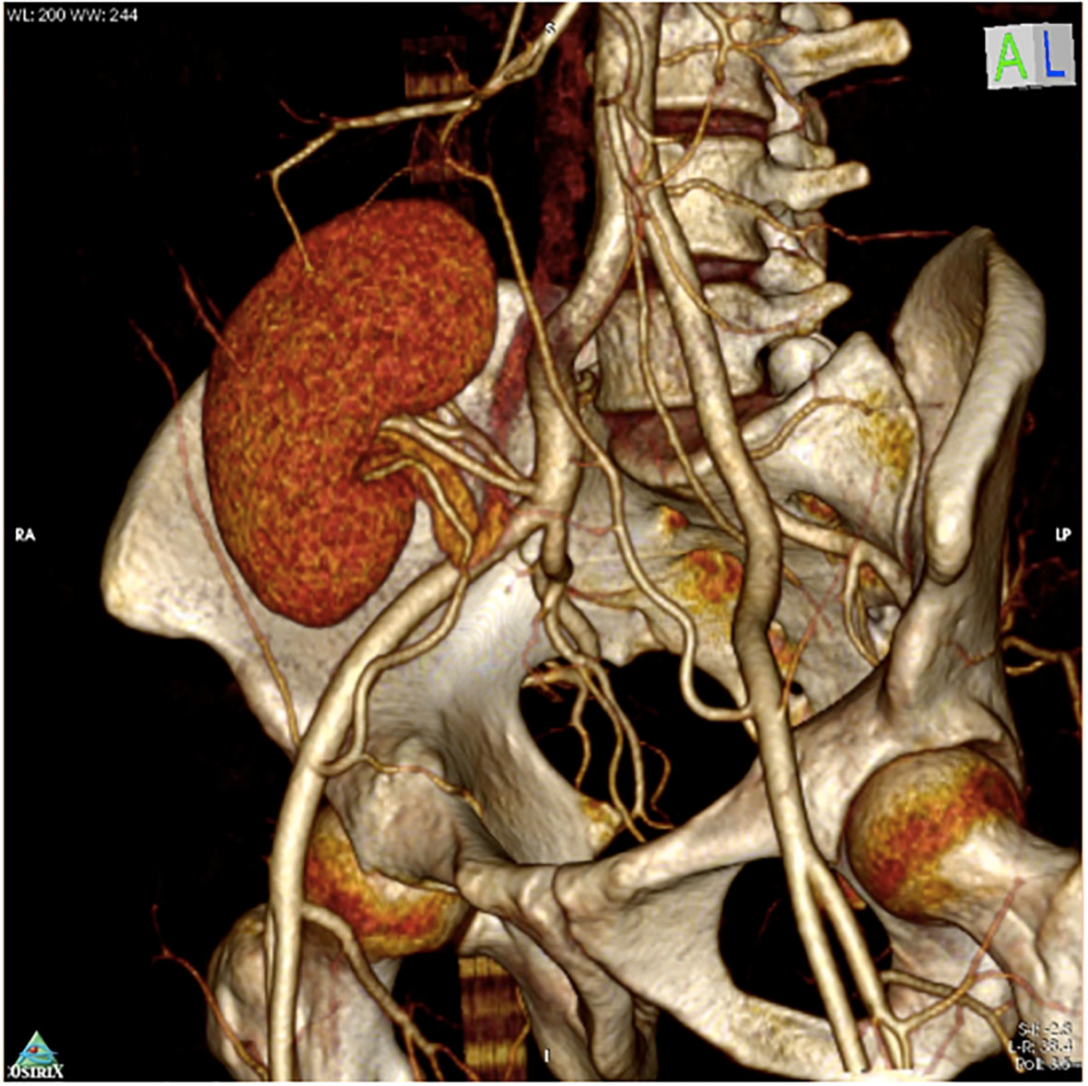
CT angiogram after 3 months after RAKT showing patent Pantaloon and accessory lower pole vessel anastomosis.

Delayed graft function (DGF) was defined as the need for dialysis within a week post renal transplantation and was managed according to the cause. USG Doppler and Tacrolimus levels were performed in all cases. Kidney biopsy was performed as indicated. CT angiography was performed to check patency in all patients with use of inferior epigastric artery in RAKT cases at 3 months (Figure 4).

### Propensity Score Analysis

Propensity score analysis was performed using nine preoperative clinical covariates: age, sex, body mass index, pre-emptive transplantation, duration of dialysis, Charlson comorbidity index, diabetes mellitus, pre-operative eGFR, and vascular anatomy as independent variables with RAKT vs. OKT as binary dependant variable by multiple logistic regression analysis. This analysis eliminated potential confounders in the data.

A 1:1 match was performed between RAKT and OKT. Nearest neighbor algorithm was used for matching. Patients were matched for any significant differences (22). This strategy allowed for the inclusion of comparable, RAKT, and OKT cohorts.

### Statistical Analysis

The preoperative, intraoperative, and postoperative parameters and functional outcomes were analyzed. Demographic and clinical data are presented as frequency distribution and simple percentages. Continuous variables are expressed as the mean ± standard deviation or the median (and interquartile ranges, IQR). Mann–Whitney test was used to compare distribution in continuous variables. The Pearson's chi-square test was used to compare the distribution for categorical variables. Kaplan–Meier curves were used to analyze graft and patient survival. The two groups were compared for graft and patient survival.

Statistical analyses were performed using SPSS v.22.0 (IBM Corp., Armonk, NY, USA) and STATA 13 (Stata Corp., College Station, TX, USA). All tests were two-sided with a significance level set at *p* < 0.05.

## Results

A total of 153 patients underwent kidney transplant with GMVs. Fifty patients underwent the procedure robotically and 103 patients underwent open kidney transplants. By matching the two approaches for the significant demographics and preoperative risk factors (>0.10 standardized difference), 86 procedures (43 in each group) were included for assessing the outcomes. The results are summarized in Tables 2–**7**.

**Table 2:** Propensity score matching (PSM) of the two groups.

**Patients**	**Unmatched comparisons**				**Matched comparisons**			
**characteristics**	**OKT** ***N =* 103**	**RAKT** ***N =* 50**	***p*-value**	Standardized difference^*^	**OKT** ***N =* 43**	**RAKT** ***N =* 43**	***p*-value**	Standardized difference^*^
Age (y), mean ± SD	43.5 ± 12.8	41.2 ± 12.6	0.7	0.02	42 ± 15	40.3 ± 13.4	0.8	0.01
Male sex, *n* (%)	77 (75)	35 (70)	0.1	−0.01	27 (65)	30 (70)	0.2	0.04
BMI, kg/m^2^								
mean ± SD	21.3 ± 4	27.3 ± 3.9	0.04	0.07	22.6 ± 4.4	26.8 ± 4.2	0.05	0.02
Charlson comorbidity index, *n* (%)								
2	65 (63)	35 (70)			27 (63)	29 (67)		
3	21 (20)	11 (22)	0.03	−0.17	09 (21)	10 (24)	0.4	0.25
4	17 (17)	04 (08)			07 (16)	04 (9)		
Diabetes Mellitus *n* (%)	19	12	0.4	0.1	6 (14)	7 (14.5)	0.9	−0.01
Preoperative eGFR (ml/min/1.73 m^2^)								
mean ± SD	10 ± 5.3	10.4 ± 4.8	0.6	0.02	11 ± 3.9	10.8 ± 4.3	0.8	0.01
Median (IQR)	9.8 (7.8-13.9)	11 (8.2–14.2)			10 (8–12.9)	11.3 (8.6–14.5)		
Pre-emptive, *n* (%)	16 (15)	12 (24)	0.5	0.15	9 (21)	10 (23.2)	0.3	0.07
Vascular anatomy	2:1 (77[75])	2:1 (41 [82])			2:1 (34[80])	2:1 (35 [82])	0.5	0.02
Arteries: Veins	3:1 (21 [20])	3:1 (6 [11])			3:1 (7 [15])	3:1 (5 [11])		
(*n*[%])	1:2 (5[5])	1:2 (3 [7])	0.7	0.2	1:2 (2 [5])	1:2 (3 [7])		
Duration of Dialysis (Days)	316 (18–455)	308 (20–500)			290 (22–360)	300 (20–470)	0.9	0.04
Side of transplant								
Left Right	2 101	050	0.4	0.01	0 43	043	0.9	0.2
Donor Characteristics								
Age (y), mean ± SD Female Sex, n (%) BMI, (mean ± SD) HLA% (mean ± SD) Left side, n (%) GFR (mean ± SD)	47 ± 12 76 (73.7) 28.3 ± 3.6 35.30 ± 29.18 93 (90.3) 95.3 ± 23.4	49 ± 12 36 (72) 29.4 ± 2.9 37.37 ± 29.49 45 (90.5) 93.6 ± 24.5	0.5 0.7 0.1 0.09 0.3 0.08	0.04 0.5 0.8 0.5 0.7 0.1	45.8 ± 11.7 32 (74.4) 28.1 ± 4.2 37.11 ± 30.52 39 (91) 96.2 ± 24.6	46.3 ± 12.8 31 (73) 28.9 ± 3.4 37.29 ± 29.56 40 (93) 95.9 ± 25.6	0.4 0.07 0.8 0.9 0.07 0.3	0.9 0.6 0.9 0.4 0.8 0.4

*Data are presented as frequencies and percentages (%) or mean ± SD*.

* *Standardized difference = difference in mean or proportions divided by the standard error; imbalance between groups was defined as absolute value >0.10 (corresponding to a small effect size)*.

### Baseline and Demographic Details

The preoperative characteristics of both the unmatched cohorts (RAKT and OKT) are given in Table 2. Overall, 103 (69%) patients were treated with OKT and 50 (31%) were treated with RAKT. There were no significant differences in preoperative clinical characteristics except for body mass index (*p* = 0.04) and Charlson comorbidity index (*p* = 0.03). Patient undergoing robotic transplants had higher BMI and lower Charlson comorbidity index. Propensity score-matched RAKT (*n* = 43) and OKT (*n* = 43) preoperative data are presented in Table 2 and are comparable.

### Intraoperative Data

There was no statistical difference in Warm ischemia time, Cold ischemia time, Re-warming time, Total Ischemia time, and anastomosis times in both groups (Tables 3, 4; Figures 7–10). The estimated blood loss (median) was 160 ml and 200 ml for RAKTs and OKTs, respectively (*p* = 0.5). The length of surgical incision was less in RAKT (*p* = 0.04) compared to OKT (median of 16 cm in OKT vs. 5.5 cm in RAKT).

**Table 3:** Intraoperative outcomes.

	**OKT**	**RAKT**	***P*-value**
Operative time (min) Median	235 (190–300)	250 (210–300)	0.6
Console time (min)	Not Applicable	170 (130–200)	
Warm ischemia time (min)	2.5 (2–4)	3 (2–5)	0.09
Cold ischemia time (min)	29 (18–35)	33 (25–40)	0.08
Rewarming time (min)	56 (48–71)	60 (50–75)	0.2
Total Ischemia time (min)	86 (65–98)	90 (70–105)	0.1
Estimated blood loss (ml)	200 (90–300)	160 (70–180)	0.5
Surgical incision length (cms)	16 (15–18)	5.5 (5–7)	0.04
Conversion	NA	0	

**Table 4:** Overall times for anastomosis during RAKT and OKT (min) (median, IQR).

**Anastomosis**	**RAKT**	**OKT**	***P*-value**
	**(*n =* 43)**	**(*n =* 43)**	
Arterial	12 (9–18)	13 (8–18)	0.7
Venous	11 (6–15)	7 (7–17)	0.6
Accessory artery	18 (12–28)	18 (13–30)	0.8
Uretero-vesical	18 (12–32)	19 (14–32)	0.7

**Figure 5:**
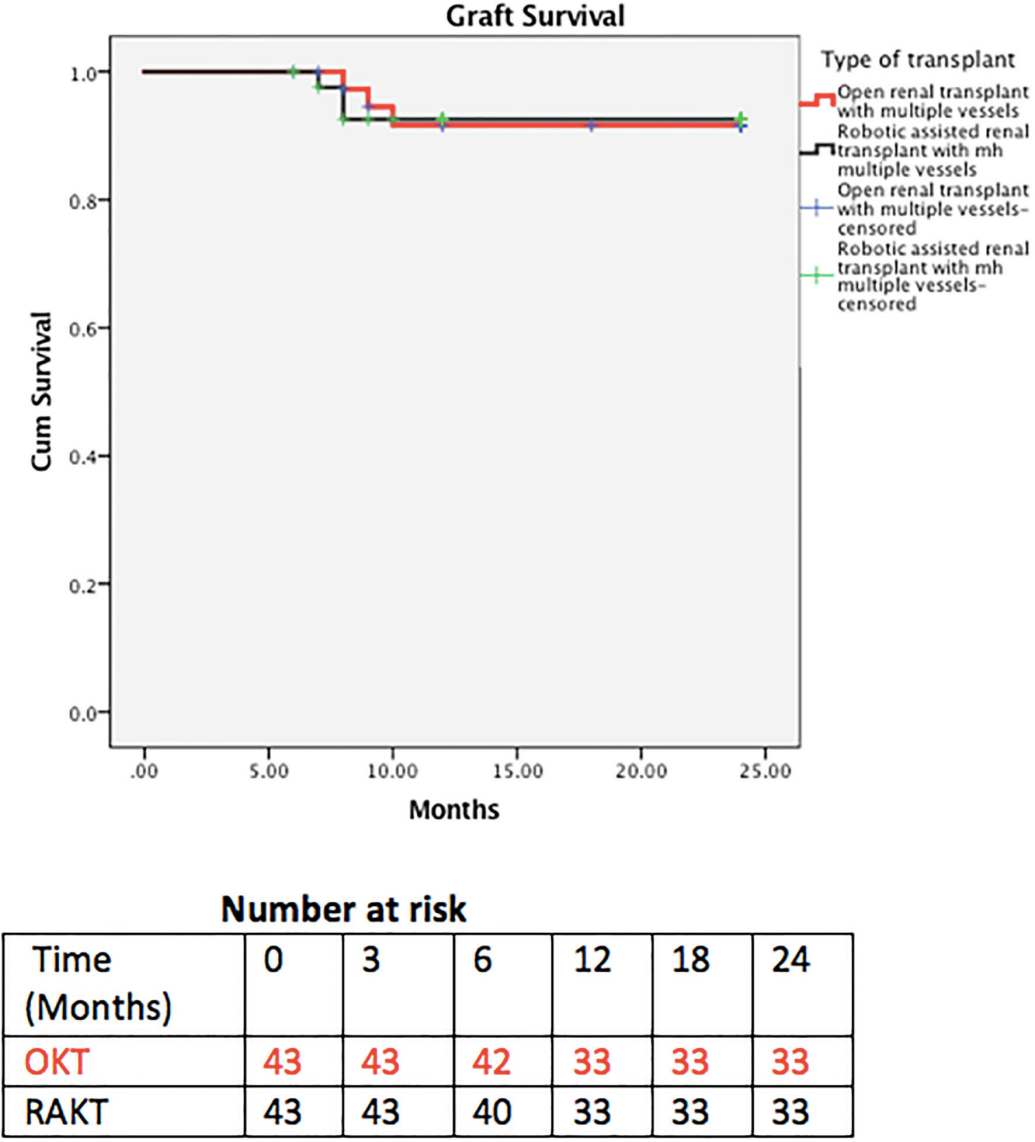
Kaplan-Meier curve of kidney allograft survival (censored for recipient death).

**Figure 6:**
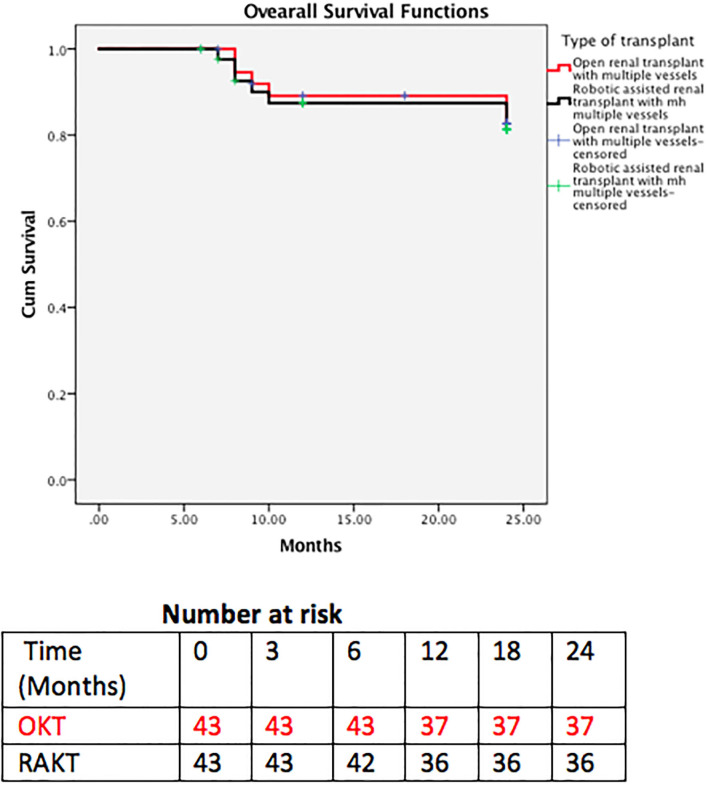
Overall survival.

**Figure 7:**
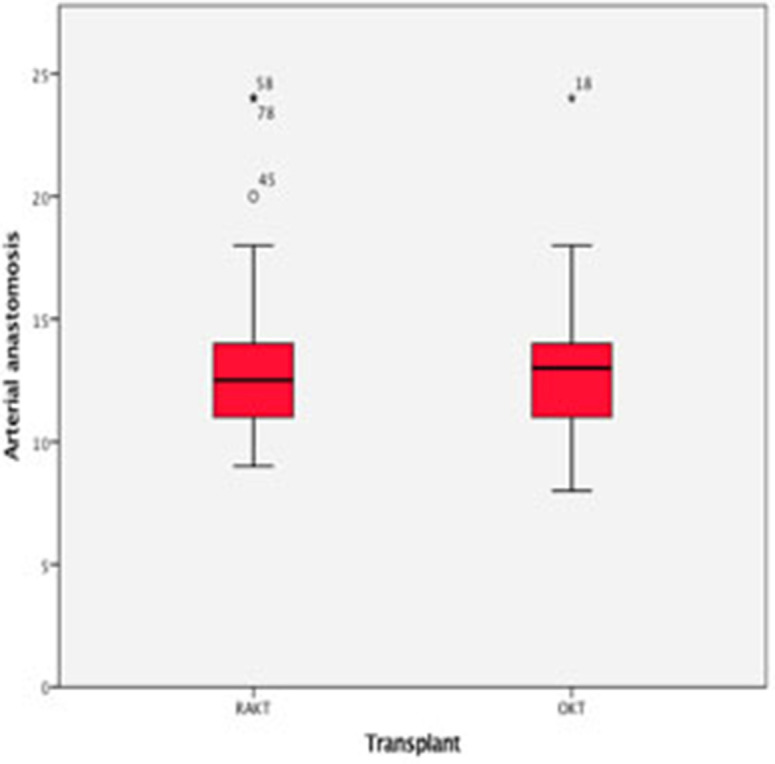
Box plot showing time for arterial anastomosis.

**Figure 8:**
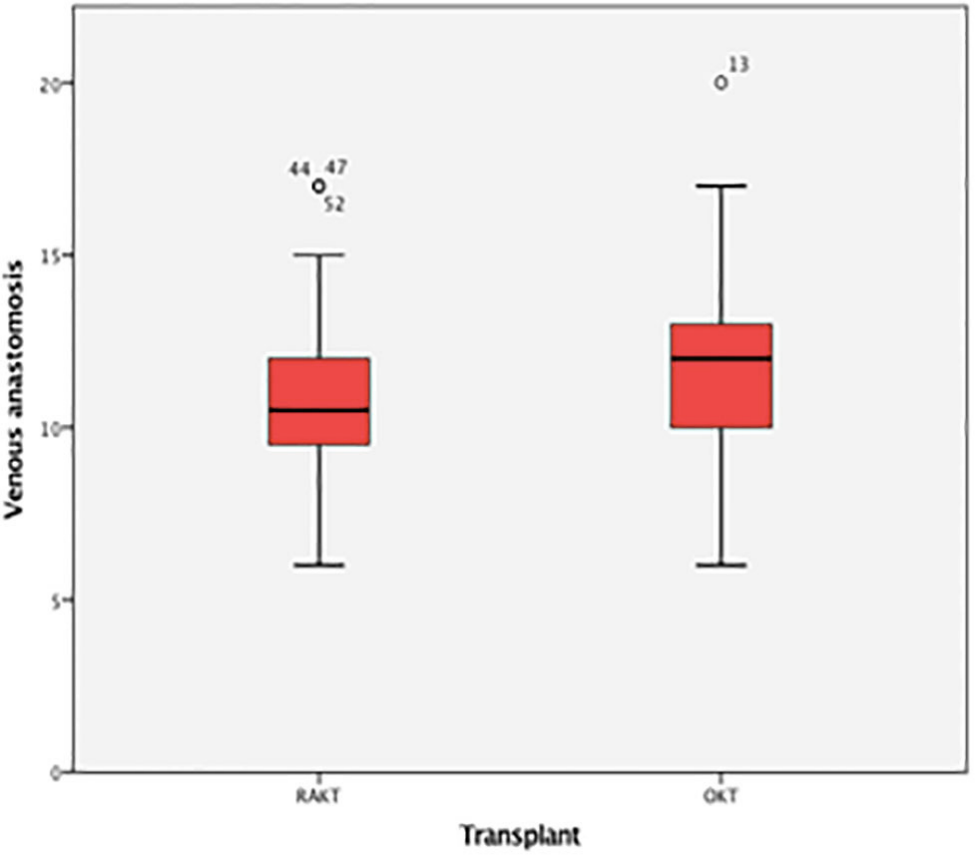
Box plot showing time for venous anastomosis.

**Figure 9:**
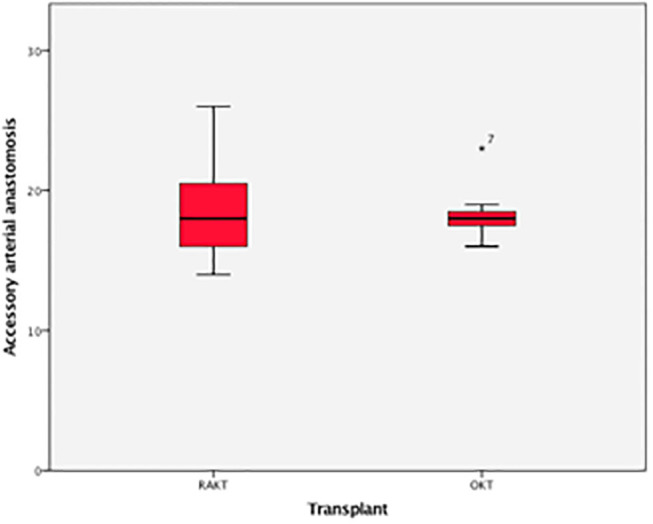
Box plot time for anastomosis of accessory artery.

**Figure 10:**
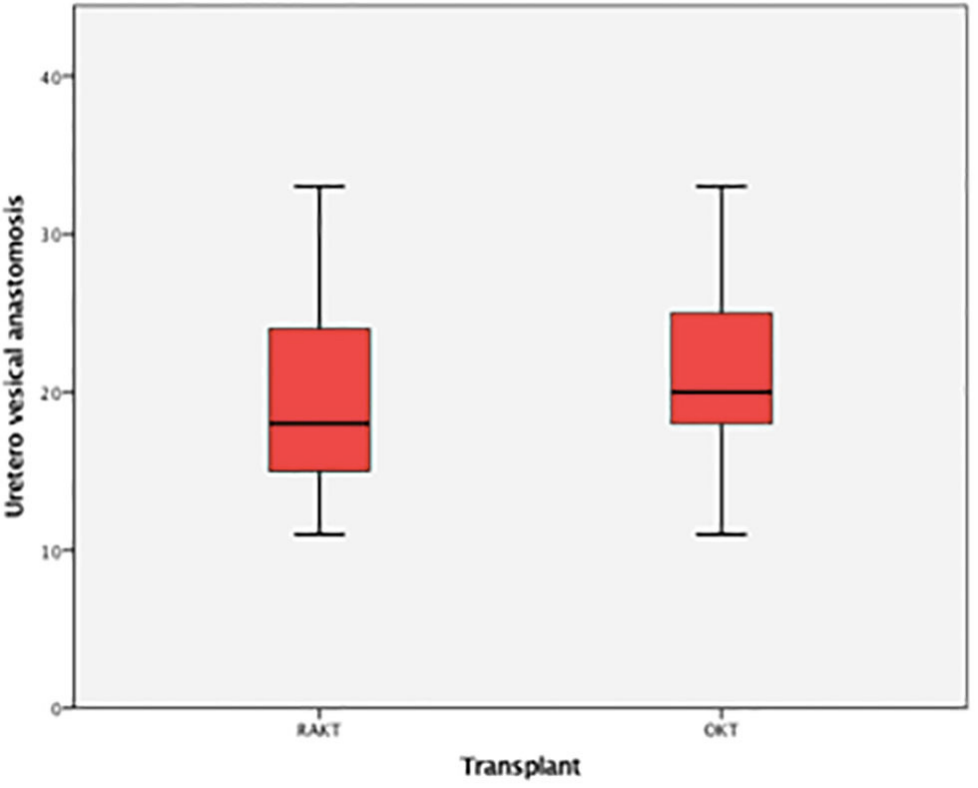
Box plot time for uretero-vesical anastomosis.

### Postoperative Course, Events, and Complications

Visual analog pain scores revealed no significant difference at 12 h but a significant difference at 24 h (*p* = 0.04) and 48 h (*p* = 0.03). The mean analgesic requirement of the RAKT group at Day 1 was less compared to OKT despite regular use of epidural anesthesia in OKT in addition (*p* = 0.02) (Table 5). RAKT cohorts had more ileus (*p* = 0.001) and less wound infection (*p* = 0.002) compared to OKT. The duration of hospital stay was longer in the OKT group at 10 (7–21) days compared to 7 (5–15) days in the RAKT group. There was no lymphocele, urinoma, or ureteral stricture in both groups (Table 6).

**Table 5:** Postoperative outcomes.

	**OKT (43)**	**RAKT (43)**	***P*-value**
Postoperative pain (VAS scale)			
12 h	6	5	0.8
24 h	5	3	0.04
48 h	4	2	0.03
Mean Analgesic requirement in grams on POD 1	4 ± 0.3	2 ± 0.15	0.02
Drain removal (median, range)	3 (2–5)	6 (4–11)	0.004
Double J stent removal (median, range)	21 (13–100)	17 (12–50)	0.09
Hospital stay	10 (7–21)	7 (5–15)	0.05
Re-admission rate	10	8	0.8
Acute rejection	3	2	0.3
UTI	3	3	0.5
Fever	4	3	0.8

**Table 6:** Modified Clavien–Dindo Grading System of complications.

**Grade**	**RAKT**	**OKT**	***P*-value**
I			
Bleeding	2	3	0.8
Wound infection	0	6	0.04
Ileus	8	1	0.03
II			
Haemorrhages requiring transfusions	3	6	0.126
IIIa			NA
Lymphoceles	0	0	
IIIb			
Graft vessel thrombosis	0	0	
Re–exploration	0	0	NA
Graft Nephrectomy	0	0	
IVa			
Delayed graft function requiring temporary dialysis	0	0	NA
Biopsy proven rejection of Rejection of graft	2	3	0.8
IVb	0	0	NA
V	0	0	NA

### Functional Outcome and Follow-Up Data

The eGFR and mean serum creatinine at discharge, and 1, 3, and 6 months were similar in both groups (Table 7). Three (6.9%) patients in RAKT and 4 (9.3%) patients in OKT had rejection confirmed on graft biopsies (*p* = 0.8).

**Table 7:** Functional outcomes.

	**OKT**	**RAKT**	***P*-value**
Median eGFR (ml/min/1.73 m^2^)			
POD 1	22.8 (13–25.7)	21.5 (16.0–28.4)	0.9
POD2	41.7 (25.0–58.0)	42.2 (30.8–53.6)	0.6
POD3	58.0 (44.0–74.0)	57.1 (41.8–66.5)	0.5
Serum Creatinine			
Mean (SD)			
At Discharge	1.46 (1.12)	1.6 (1.32)	0.6
At 1 month	1.39 (1.30)	1.5 (1.40)	0.5
At 3 months	1.14 (1.03)	1.25 (1.42)	0.2
At 6 months	1.07 (0.90)	1.12 (1.03)	0.4
Mean e GFR at 6 months (death censored) ml/ min/1.73 m^2^	61.8 ± 16	62.4 ± 24	0.9
Delayed Graft function, *n* (%)	0	0	

### Long Term Outcome

Patient and graft survival were comparable in the two groups with 93 and 92.5% 2 year graft survival in RAKT and OKT (Figure 5), respectively (log rank test *p* = 0.947). Two-year patient survival was 83.7 and 85% for RAKT and OKT (Figure 6), respectively (log rank test *p* = 0.844).

## Discussion

Since inception of RAKT by Giulianotti et al. (23) followed by improvement in the technique by the Vattikuti-Medanta team (11, 12), there have been various publications of RAKT series. However, to the best of our knowledge, studies comparing RAKT with OKT in GMVs in comparable cohorts are sparse.

In the present study, we found no statistical difference in ischemia times, operative duration, anastomotic times, blood loss, serum creatinine, and eGFR at discharge and at 6 months between RAKT and OKT cohorts using GMVs. Due to lesser incidence of wound-related events, RAKT has emerged as an attractive option in obese patients (24–26). This is due to the smaller non-muscle cutting periumbilical incision having least fat between skin and fascial layers underneath, and the nature of minimally invasive surgery. Of note, rates of wound infection were lesser in the RAKT group (*p* = 0.002) in our series. The length of surgical incision, visual analog pain score, requirement of analgesia, and the duration of hospital stay were also less in the RAKT group, imparting minimally invasive benefits to this group.

Due to the extraperitoneal approach in OKT, drains are kept for a longer time to prevent formation of lymphoceles. RAKT offers the advantage of early drain withdrawal due to the transperitoneal nature of surgery. The drains were removed at a median (range) of 3 (2–5) days and 6 (4–11) days in RAKT and OKT groups, respectively. Lymph drainage is absorbed from the peritoneum, so drain may be removed as soon as one makes sure of absence of urine leak by performing drain fluid creatinine in transperitoneal RAKT group, which enables patients to be discharged earlier. In the OKT group, drains were removed later, only after drain amount is <50 ml, because of the potential risk of lymphoceles if removed earlier. However, there was no evidence of lymphoceles in both our groups.

The rate of ureteric complications described in literature is around 3% in GMVs (9, 10). Interestingly, in our study, we did not have any urinoma or ureteric stricture and there was no need to perform pyelo-uretric anastomosis due to devascularized ureter.

Robotic surgery provides 10× magnification and enables access to parts of the body with six degrees of freedom, with additional tremor filtration (21). These help an experienced surgeon to perform vascular anastomoses of small vessels like polar vessels and inferior epigastric vessels.

RAKT has proven to be equivalent to OKT with the added advantage of it being minimally invasive (11, 12). The transplant guidelines provided by European Association of Urology recommend that grafts with multiple renal arteries can be used for live renal transplantation (15). Conventionally, OKT is preferred in transplanting GMVs (17). With increased acceptance of RAKT and growing experience in this field, graft with multiple renal vessels should be accepted and not considered a contraindication for RAKT (16).

The present study demonstrates the feasibility of RAKT using GMVs from living donors. We have described a standardized operative protocol for RAKT using GMVs by adopting vascular reconstruction techniques similar to OKT with GMVs (Table 1). Both arterial and venous anastomosis could be performed without any conversions.

The study has limitations. First, our study was a retrospective analysis of prospectively collected data and we attempt to take care of the inclusion bias with propensity score matching. Second, the sample size was small. Third, the study evaluated only short- to mid-term perioperative outcomes. Despite all the limitations, this study paves a big step forward toward developing the surgical technique in RAKT with GMVs with outcomes similar to OKT.

## Conclusion

GMVs in RAKT have comparable patient and graft survival similar to OKT with GMVs. Satisfactory functional outcome can be achieved by RAKT similar to OKT in GMVs. RAKT seems to have advantage over OKT in that it is minimally invasive with fewer complications. Larger studies with longer follow-up are needed.

## Data Availability Statement

All datasets generated for this study are included in the article/supplementary material.

## Author Contributions

SN: protocol development, data collection, data analysis, and manuscript writing. FZ: data collection. PG: manuscript editing. RA: protocol development, data analysis, and manuscript editing. All authors read and approved the final manuscript.

## Conflict of Interest

The authors declare that the research was conducted in the absence of any commercial or financial relationships that could be construed as a potential conflict of interest.

## References

[1] Oesterwitz H Strobelt V Scholz D Mebel M. Extracorporeal microsurgical repair of injured multiple donor kidney arteries prior to cadaveric allotransplantation. Eur Urol. (1985) 11:100–5. 10.1159/000472465 3891354

[2] Bouchou F Kamel G Gelet A Devonec M Mongin D Traeger J Transplantation of kidneys with multiple renal arteries. Transplant Proc. (1984) 16:273.

[3] Emiroglu R Koseoglu F Karakayali H Bilgin N Haberal M. Multiple artery anastomosis in kidney transplantation. Transplant Proc. (2000) 32:617–19. 10.1016/S0041-1345(00)00919-2 10812141

[4] Roza AM Perloff LJ Naji A Grossman RA Barker CF. Living-related donors with bilateral multiple renal arteries. A twenty-year experience. Transplantation. (1989) 47:397–9. 10.1097/00007890-198902000-00045 2645725

[5] Guerra EE Didone EC Zanotelli ML Vitola SP Cantisani GP Goldani JC. Renal transplants with multiple arteries. Transplant Proc. (1992) 24:1868. 1412889

[6] Pourmand G Mehraban D Ameli PJ Ayati M Naderi G. Donor polar kidney arteries: experience with 10 cases among 140 living-related kidney transplants. Transplant Proc. (1992) 24:1867. 1412888

[7] Benedetti E Troppmann C Gillingham K Sutherland DE Payne WD Dunn DL. Short-and long-term outcomes of kidney transplants with multiple renal arteries. Ann Surgery. (1995) 221:406–14. 10.1097/00000658-199504000-00012 7726677 PMC1234591

[8] Kumar A Gupta RS Srivastava A Bansal P. Sequential anastomosis of accessory renal artery to inferior epigastric artery in the management of multiple arteries in live related renal transplantation: a critical appraisal. Clin Transplant. (2001) 15:131–5. 10.1034/j.1399-0012.2001.150209.x 11264640

[9] Afriansyah A Rasyid N Rodjani A Wahyudi I Mochtar CA Susalit E. Laparoscopic procurement of single versus multiple artery kidney allografts: meta-analysis of comparative studies. Asian J Surg. (2019) 42:61–70. 10.1016/j.asjsur.2018.06.001 30042021

[10] Carter JT Freise CE McTaggart RA Mahanty HD Kang SM Chan SH. Laparoscopic procurement of kidneys with multiple renal arteries is associated with increased ureteral complications in the recipient. Am J Transplant. (2005) 5:1312–8. 10.1111/j.1600-6143.2005.00859.x 15888035

[11] Menon M Abaza R Sood A Ahlawat R Ghani KR Jeong W. Robotic kidney transplantation with regional hypothermia: evolution of a novel procedure utilizing the IDEAL guidelines (IDEAL phase 0 and 1). Eur Urol. (2014) 65:1001–9. 10.1016/j.eururo.2013.11.011 24287316

[12] Menon M Sood A Bhandari M Kher V Ghosh P Abaza R Robotic kidney transplantation with regional hypothermia: a step-by-step description of the Vattikuti Urology Institute-Medanta technique (IDEAL phase 2a). Eur Urol. (2014) 65:991–1000. 10.1016/j.eururo.2013.12.006 24388099

[13] Siena G Campi R Decaestecker K Tugcu V Sahin S Alcaraz A. Robot assisted kidney transplantation with regional hypothermia using grafts with multiple vessels after extracorporeal vascular reconstruction: results from the European Association of Urology Robotic Urology Section Working Group. Eur Urol Focus. (2018) 4:175–84. 10.1016/j.euf.2018.07.022 30049659

[14] Breda A Territo A Gausa L Tugcu V Alcaraz A Musquera M. Robot-assisted kidney transplantation: the European experience. Eur Urol. (2018) 73:273–81. 10.1016/j.eururo.2017.08.028 28916408

[15] Karam G Kälble T Alcaraz A European Association of Urology (EAU) Guidelines on kidney transplantation. Version 2014. (2014). Available online at: http://uroweb.org/guideline/renal-transplantation/ (accessed March 24, 2020).

[16] Kishore TA Kuriakose MJ Pathrose G Raveendran V Kumar KV Unni VN. Robotic assisted kidney transplantation in grafts with multiple vessels: single centre experience. Int Urol Nephrol. (2020) 52:247–52. 10.1007/s11255-019-02305-z 31586280

[17] Ghazanfar A Tavakoli A Zaki MR Pararajasingam R Campbell T Parrott NR. The outcomes of living donor renal transplants with multiple renal arteries: a large cohort study with a mean follow-up period of 10 years. Transplant Proc. (2010) 42:1654–8. 10.1016/j.transproceed.2009.12.067 20620494

[18] Novick AC. Microvascular reconstruction of complex branch renal artery disease. Urol Clin N Am. (1984) 11:465. 6380079

[19] Kok NF Dols LF Hunink MG Alwayn IP Tran KT Weimar W. Complex vascular anatomy in live kidney donation: imaging and consequences for clinical outcome. Transplantation. (2008) 85:1760–5. 10.1097/TP.0b013e318172802d 18580468

[20] Sood A Ghosh P Jeong W Khanna S Das J Bhandari M. Minimally invasive kidney transplantation: perioperative considerations and key 6-month outcomes. Transplantation. (2015) 99:316–23. 10.1097/TP.0000000000000590 25606784

[21] Tugcu V Sener NC Sahin S Yavuzsan AH Akbay FG Apaydin S. Robot-assisted kidney transplantation: comparison of the first 40 cases of open vs robot-assisted transplantations by a single surgeon. BJU Int. (2018) 121:275–80. 10.1111/bju.14014 28921838

[22] Austin PC An introduction to propensity score methods for reducing the effects of confounding in observational studies. Multivar Behav Res. (2011) 463:399–424. 10.1080/00273171.2011.568786 PMC3144483 21818162

[23] Giulianotti P Gorodner V Sbrana F Tzvetanov I Jeon H Bianco F. Robotic transabdominal kidney transplantation in a morbidly obese patient. Am J Transplant. (2010) 10:1478–82. 10.1111/j.1600-6143.2010.03116.x 20486912

[24] Tzvetanov I D'Amico G Benedetti E. Robotic-assisted kidney transplantation: our experience and literature review. Curr Transplant Rep. (2015) 2:122–6. 10.1007/s40472-015-0051-z 26000230 PMC4431703

[25] Tzvetanov I Giulianotti PC Bejarano-Pineda L Jeon H Garcia-Roca R Bianco F. Robotic-assisted kidney transplantation. Surg Clin North Am. (2013) 93:1309–23. 10.1016/j.suc.2013.08.003 24206853

[26] Garcia-Roca R Garcia-Aroz S Tzvetanov I Jeon H Oberholzer J Benedetti E Single center experience with robotic kidney transplantation for recipients with BMI of 40 kg/m^2^ or greater: a comparison with the UNOS Registry. Transplantation. (2017) 101:191–6. 10.1097/TP.0000000000001249 27152921

